# Enhanced recovery in colorectal surgery: a multicentre study

**DOI:** 10.1186/1471-2482-11-9

**Published:** 2011-04-14

**Authors:** José M Ramírez, Juan A Blasco, José V Roig, Sergio Maeso-Martínez, José E Casal, Fernando Esteban, Daniel Callejo Lic

**Affiliations:** 1 Department of Colorectal Surgery, Hospital Clínico Universitario Lozano Blesa, Zaragoza, Spain; 2 Health Technology Assessment, Agencia Laín Entralgo, Madrid, Spain; 3 Department of Surgery, Hospital General Universitario de Valencia, Valencia, Spain; 4 Department of Surgery, Hospital Do Meixoeiro, Vigo, Spain; 5 Department of Surgery, Hospital Clínico San Carlos, Madrid, Spain

## Abstract

**Background:**

Major colorectal surgery usually requires a hospital stay of more than 12 days. Inadequate pain management, intestinal dysfunction and immobilisation are the main factors associated with delay in recovery. The present work assesses the short and medium term results achieved by an enhanced recovery program based on previously published protocols.

**Methods:**

This prospective study, performed at 12 Spanish hospitals in 2008 and 2009, involved 300 patients. All patients underwent elective colorectal resection for cancer following an enhanced recovery program. The main elements of this program were: preoperative advice, no colon preparation, provision of carbohydrate-rich drinks one day prior and on the morning of surgery, goal directed fluid administration, body temperature control during surgery, avoiding drainages and nasogastric tubes, early mobilisation, and the taking of oral fluids in the early postoperative period. Perioperative morbidity and mortality data were collected and the length of hospital stay and protocol compliance recorded.

**Results:**

The median age of the patients was 68 years. Fifty-two % of the patients were women. The distribution of patients by ASA class was: I 10%, II 50% and III 40%. Sixty-four % of interventions were laparoscopic; 15% required conversion to laparotomy. The majority of patients underwent sigmoidectomy or right hemicolectomy. The overall compliance to protocol was approximately 65%, but varied widely in its different components. The median length of postoperative hospital stay was 6 days. Some 3% of patients were readmitted to hospital after discharge; some 7% required repeat surgery during their initial hospitalisation or after readmission. The most common complications were surgical (24%), followed by septic (11%) or other medical complications (10%). Three patients (1%) died during follow-up. Some 31% of patients suffered symptoms that delayed their discharge, the most common being vomiting or nausea (12%), dyspnoea (7%) and fever (5%).

**Conclusion:**

The following of this enhanced recovery program posed no risk to patients in terms of morbidity, mortality and shortened the length of their hospital stay. Overall compliance to protocol was 65%. The following of this program was of benefit to patients and reduces costs by shortening the length of hospital stay. The implantation of such programmes is therefore highly recommended.

## Background

Major colorectal surgery, i.e., surgery that involves wide resection of the colon and anastomosis, generally involves a prolonged hospital stay - on average 12-14 days. A stay of one week is usually the minimum that can be expected [1]. This prolonged occupation of a hospital bed is not usually owed to problems of morbidity but to the conventional care protocol followed. For decades this protocol has hardly been modified: it therefore does not take into account the advances that have been made in the perioperative management of such patients.

Inadequate pain management, intestinal dysfunction and immobilisation have been recognised since at least 1997 as among the main factors delaying postoperative recovery in patients subjected to major surgery [2]. This led Kehlet et al [3] to propose a series of measures designed to improve recovery following major colorectal surgery in their well-known multimodal recovery program. Currently, evidence-based multidisciplinary action protocols of this kind, known initially as fast-track or better called enhanced recovery programs (ERAS), are not achieving the degree of implantation hoped for [3]. Some authors suggest this to be due to the organisational demands they make on surgeons, anaesthetists and nursing staff, and to social, cultural and economic realities [4].

The results that can be achieved with ERAS - reductions in postoperative morbidity, average length of hospital stay and the consumption of resources - are, however, significant, and the general implantation of an ERAS for patients who are to undergo colorectal surgery is recommendable [5-8]. In some of the clinical practice guides available at http://www.reducinglengthofstay.org/, these programmes are considered to represent best clinical practice according to current scientific evidence. Unfortunately, the results communicated regarding surgery in an ERAS context have nearly all come from individual institutions, although they include those of four randomised clinical trial [5,9-11]. Recently the results of an international study (five hospitals in different countries, four with no prior experience in ERAS) reporting on patient follow-up, the degree of acceptance and the degree of compliance with the protocol of a common ERAS, have also become available [12]. The results of these studies [5,9-12] suggest that just making a protocol available is insufficient for objectives to be achieved; changes also need to be made to organisational strategies and the medical professionals involved in pre, intra and especially postoperative care require support, perhaps via continuing education.

The present work analyses the short and medium term results returned by an ERAS for colorectal surgery based on previously published protocols, followed at 12 Spanish hospitals.

## Methods

### Participating centres

The twelve participating centres were chosen for their organisational abilities and their experience and interest in colorectal surgery and patient care. These centres, distributed around Spain, ranged from large university hospitals to medium and small area hospitals (the Clínico from Zaragoza, La Paz, Clínico San Carlos and Gregorio Marañón from Madrid, General from Valencia, Mútua Terrassa, Do Meixoeiro from Vigo, Hospital d'Igualada, La Mancha Centro from Alcázar de San Juan, Universitario de Elche, Son Llatzer from Palma de Mallorca and Fundación Calahorra hospitals) with the collaboration of Health Technology Assessment Unit from Agencia Laín Entralgo, Madrid.

There were two meetings with at least two professionals of each centre, a surgeon and an anaesthetist. During these meetings discussions were held with national and international experts who assisted the group in the implementation of the program. These professionals were the persons in charge to develop the program in their centre and to starting the implementation of the protocol in two months after the second work session. There were two annual reunions with the group to supervise and improve the compliance of the protocol.

### Study design

This prospective study, which involved 300 patients, was performed between 2008 and 2009. The ERAS used was developed by the authors on the basis of published protocols [3,5,8-12]. The variables recorded included perioperative mortality, length of hospital stay and compliance with the protocol.

The study was presented to the Hospital Ethical Board and accepted as this is an observational non-randomised study based on the best available evidence.

The research was conducted conformed to the Helsinki Declaration and to local legislation. Patients gave informed consent to participate in the study. This study has been registered in the ISRCTN register with the number ISRCTN16397735, you can also access to the registration information by the URL: http://www.controlled-trials.com/ISRCTN16397735.

### Inclusion and exclusion criteria

A) Inclusion criteria. All patients had to be 18 years of age or over and to be programmed for surgery for colorectal cancer without the need for a stoma or any further surgical procedure.

B) Exclusion criteria: The need for emergency surgery, an American Society of Anaesthesiologists (ASA) class of IV, the need for a colostomy or ileostomy, the inability to provide informed consent, diabetes, slow evacuation previously documented by a digestive medicine unit.

### Enhanced recovery program protocol

The ERAS required that, during the preoperative period, patients be given advice, that there be no preparation of the colon and that patients receive four carbohydrate-rich drinks (4 × 200 ml) one day prior to surgery plus two further drinks (2 × 200 ml) on the morning of surgery. During surgery, goal directed fluids were administered using oesophageal Doppler monitoring, and hypothermia and drainages avoided. After surgery, nasogastric tubes were not used, early mobilisation was practised, and oral fluids administered early. Table 1 summarises the protocol characteristics of the ERAS followed.

**Table 1:** Protocol characteristics of the followed enhanced recovery program

Time	Procedure
Preoperative	a. Provision of verbal and written information to patients regarding the **ERAS**. Collection of signed consent.
	b. Malnourished patients to receive hyperproteic supplement at least twice per day during the week before surgery.

Day before surgery	a. No colon preparation.
	b. Normal food in the morning. Liquids on demand during the evening. Four bricks of carbohydrate-rich Nutricia Preop^® ^to be taken during the evening (total: 800 ml).
	c. Prophylaxis for pulmonary thromboembolism following normal practice.
	d. Antibiotic prophylaxis following normal practice.

Day of surgery (before surgery)	a. Two hours before surgery: provision of two bricks of Nutricia Preop^® ^(total: 400 ml).
	b. Antibiotic prophylaxis.

Operating room	A. Surgeons: no drainage; nasogastric tube, if needed, to be removed before extubation; if possible use transverse or curved incisions in open surgery.
	B Anaesthetists:
	-Maintenance: Oxygen/air with FiO2 >80%.
	-Monitoring: routine. Only use arterial/central catheter if unavoidable.
	-Fluids: maintenance with Hartmann (5 cc/kg/h). Bolus of gelofusine (250 cc).
	Maintain Hb > 8.0 g/dl.
	- Optimise stroke volume via oesophageal Doppler:
	-250 cc of fluid in bolus; if SV > 10% repeat until this figure is not reached. Provide no further bolus unless SV falls or there is blood loss.
	-If hypotension remains after SV correction, use a vasoconstrictor.
	-Consider use of inotropic agents if peak velocity descends and clinical signs suggest ventricular function deficit.
	-Temperature: use liquid heater and heating blanket.

Day of surgery (recovery room)	-Mask with high oxygen flow for 2 h independent of saturation. Follow with nasal cannulae to maintain SpO2 > 95%.
	-Maintain mean blood pressure >65 mmHg. If blood pressure low provide 250 cc gelofusine and reassess.

Day of surgery (ward)	-In the evening sit patient in seat for at least 2 h.
	-Liquid diet (800-1000 ml). Include two bricks of high protein/high calorie hospital dietary preparation (specific for postoperative period).
	-Minimum diuresis (500 cc in first 24 h).
	-Analgesia: 1 g paracetamol/6 h.

Postoperative day 1	-Liquid diet, at least 2 l, including 3 bricks of high protein/high calorie hospital dietary preparation.
	-Mobilisation; patient seated at least 6 h per day.
	-Suspend IV fluid if tolerated. Heparin injection for maintaining patency of intermittent infusion devices.
	-Maintain epidural analgesia pump if one in place.
	-Paracetamol 1 g/6 h.
	-Lactulose 1 sachet/12 h (preferably magnesium-based).
	-Assess meeting of discharge criteria: only oral analgesia, mobilisation reaching presurgical level, toleration of solid food, gases passed, stools passed, no nausea, and patient agrees to discharge.

Postoperative day 2	-Suspend epidural catheter; begin with NSAIDS; diet soft/normal; mobilisation on demand; remove urinary catheter and assess meeting of discharge criteria.

Postoperative day 3	-Check general status; assess meeting of discharge criteria and take decision in this respect.

Follow up	-Telephone monitoring for 48 h.
	-First out-patient visit 10-14 days after surgery.

SV: stroke volume; IV: intravenous; Hb: haemoglobin.

Although not explicitly required by the ERAS, the following variables were also recorded: opioid-free pain control, the use of prophylactic medication for postoperative vomiting and nausea, and the use of epidural anaesthesia.

Patients were discharged following the criteria established by the ERAS protocol. All patients were followed-up for at least three months.

An online database http://www.ftsurgery.com/ was prepared for the collection of data from the different centres.

### Data analysis

Dichotomous variables were recorded as absolute frequencies (number of cases) and relative frequencies (percentages). It should be noted that this work reports frequencies and percentages with respect to the data made available; therefore they do not always refer to all patients. Continuous variables were recorded as means and standard deviations (SD) or median plus maximum and minimum values, depending on whether or not their distribution was normal (determined by the Kolmogorov-Smirnov test). All analyses were made using 18 version SPSS software.

## Results

### Patients, surgery and postoperative treatment

Of all patients who initially met criteria for inclusion, 16 were excluded, 9 patients were in intensive care at the discretion of the anaesthetist and 7 received an unscheduled ileostomy. A total of 300 patients were finally included.

Table 2 shows the characteristic of the patients included, the surgical techniques used and the surgical procedures followed.

**Table 2:** Patient characteristics and surgical techniques and procedures followed

Patients characteristics (n = 300)	Values	
Age (years)	69	35-88

Sex		

Female	152	51.5

Male	143	48.5

Preoperative stay (days)	0.82	1.93

Surgical risk: ASA		

I	30	10.2

II	148	50.2

III	117	39.7

Surgical technique		

Laparoscopy	171	63.6

Conventional	98	36.4

Surgical procedure		

Right hemicolectomy	98	35.6

Sigmoidectomy	74	26.9

Anterior resection	67	24.4

Left hemicolectomy	27	9.8

Transverse resection	5	1.8

Subtotal colectomy	4	1.5

Quantitative variables are expressed as medians plus minimum and maximum values; qualitative variables are expressed as absolute numbers and percentages. Preoperative stay is expressed as mean and standard deviation.

The median age of the patients was 69 years (35-88); 51.5% were women (152 patients). The patients were distributed by ASA class as follows: I 10.2% (30), II 50.2% (148) and III 39.7% (117).

Some 63.6% (171) of the patients underwent laparoscopic surgery and 36.4% (98) open surgery. Some 15.3% of the laparoscopically intervened patients required conversion to laparotomy (22).

Sigmoidectomy and right hemicolectomy made up the majority of procedures performed (62.5%). Some 5.2% of patients (15) were subjected to preoperative radiotherapy. Laparotomy was medial in 88.8% and transversal in 11.2% of patients so intervened.

The mean preoperative hospital stay was 0.82 days (SD: 1.93). The mean duration of surgery was 155.4 min. The median length of time spent in the recovery room was 240 min (60-1500).

### Main features of the protocol and their compliance rates

Table 3 shows the compliance rates for the main features outlined in the ERAS protocol, as well as for the other features not explicitly included in the protocol.

**Table 3:** Compliance with perioperative treatment and postoperative recovery after colon resection within the context of the enhanced recovery program followed

Perioperative treatment variable mentioned in ERAS protocol		
	**Number**	**Percentage**

Preoperative		

Perioperative information	290	99

No colon preparation	246	82.8

Carbohydrate-rich drinks on day before surgery^1^	187	65.2

Carbohydrate -rich drinks before surgery^2^	182	63

Surgery		

Goal directed fluids (Cardio-Q)^3^	138	46.3

No hypothermia	242	84.3

No drainage	146	53.3

Postoperative		

No nasogastric tube	222	77.1

Early mobilisation	128	44.6

Early taking of fluids by mouth^4^	116	40.6

		
**Variable not explicitly mentioned in ERAS protocol**		

	**Number**	**Percentage**

Opioid-free pain control^5^	206	78.3

Prophylactic medication for nausea and vomiting	76	26.8

Epidural anaesthesia	106	38.8

1: 40.3% received two, 59.7% received three or four; 2: 14.8% received one, 85.2% received two; 3: Mean fluid volume received was 1742.5 ml; 4: 46.8% received one, 50% received two, and 3.2% received more than two; 5: 42.8% of patients used patient controlled analgesia.

The overall compliance to protocol was approximately 65%, but varied widely in its different components.

### Postoperative hospital stay and readmission rate

**Figure 1:**
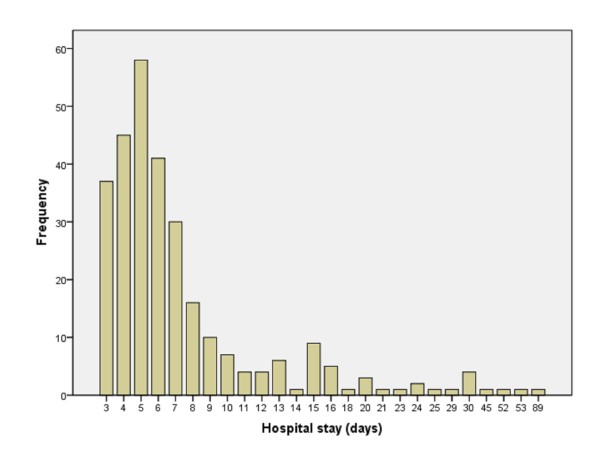
**Length of postoperative stay of patients who underwent colon surgery within the context of the present enhanced recovery program**.

The median length of postoperative hospital stay was 6 days (3-89 days). Figure 1 shows the distribution of length of postoperative stay. The mean postoperative length of stay according to surgical procedure was: right hemicolectomy and sigmoidectomy 8 days, left hemicolectomy 6 days, and transverse resection and subtotal colectomy 5 days.

Some 2.7% of the patients (8) were readmitted following discharge for medical or surgical reasons. Three patients re-presented with febrile syndrome, two with nosocomial pneumonia, and three with pulmonary thromboembolism, diarrhoea with hyponatremia or abdominal wall abscess.

Some 7% of the patients (21) required repeat surgery. The causes included dehiscence (12 patients), fistula or anastomatic leakage (three patients), evisceration (two patients), and abdominal pain, haemoperitoneum, ischemia and intestinal occlusion (one patient each). The median post-repeat-operative length of hospital stay was 6 days (1-20).

### Postoperative morbidity/mortality

Table 4 summarises the complications encountered. The most common were surgical (23.7% of patients; 71), followed by septic 11% (33) and other medical complications 9.7% (29). The most common surgical complications were wound infection (12%; 36), paralytic ileus (10%; 30; median paralysis time 3 days [2-8]), anastomosis leakage (4.3%; 13) and perioperative haemorrhage (1.7%; 5). The most common medical complications were respiratory distress (2.7%; 8), respiratory failure (2.7%%; 8) and cardiac arrhythmia or ischemia (1.7%; 5). Finally, the most common septic complications were abdominal abscess (4.3%; 13), urine infection (3.7%; 11), respiratory infection (3.7%; 11) and peritonitis (2.3%; 7).

**Table 4:** Local and general morbidity following colon resection within the enhanced recovery program followed

Total (n = 300)	Number	Percentage
Surgical complications	71	23.7

Wound infection	36	12

Paralytic ileus	30	10

Anastomosis leakage	13	4.3

Perioperative haemorrhage	5	1.7

Evisceration	3	1

Perforation	0	0

Other surgical complications	10	3.3

Septic complications	33	11

Abdominal abscess	13	4.3

Urine infection	11	3.7

Respiratory infection	11	3.7

Peritonitis	7	2.3

Sepsis	3	1

Catheter sepsis	2	0.7

Necrotising fascitis	0	0

Other septic complications	3	1

Medical complications	29	9.7

Respiratory distress	8	2.7

Respiratory failure	8	2.7

Cardiac complication	5	1.7

Pulmonary oedema	3	1

Acute urine retention	3	1

Pulmonary embolism	1	0.3

Myocardial infarction	0	0

Cerebrovascular accident	0	0

Venous thrombosis	0	0

Other medical complications	10	3.3

All complications	89	29.7

Mortality	3	1

Note: some patients presented more than one symptom.

Three patients (1%), all of whom required repeat surgery, died. Two of these died during their hospital stay due to multi-organ failure and sepsis, and one from cancer following discharge.

### Symptoms delaying discharge

Table 5 records the symptoms that delayed discharge. Some 31.3% (94) of all patients presented some such symptom, 22% (66) suffered one, 6.3% (19) suffered two, 2.7% (8) suffered three, and 0.3% (1) suffered four. The most common symptoms were vomiting (12%; 36), dyspnoea (6.7%; 20) and fever of unknown origin (4.7%; 14).

**Table 5:** Symptoms delaying discharge

Total (n = 300)	Number of patients	Percentage
Vomiting or nausea	36	12

Fatigue	20	6.7

Fever of unknown origin	14	4.7

Constipation	9	3

Dizziness	6	2

Pain	6	2

Urine retention	5	1.7

Depression, confusion	4	1.3

Diarrhoea	3	1

Scant diuresis	3	1

Other symptoms	26	8.7

Total	94	31.3

Note: some patients presented more than one symptom.

## Discussion

Each of the steps outlined in the present ERAS is based on scientific evidence. For example, patient education is reported to be important in the response to surgery. Now-classic studies [3] have shown that informed patients require less analgesia in the postoperative period and indeed experience significantly less pain than uninformed patients. More recent work has shown that adequate preoperative information reduces patient anxiety before surgery and may also hasten postsurgical recovery [3,13,14].

A number of studies on programmed colon surgery have brought into doubt the need for preoperative mechanical cleansing of the intestine [3,15]. The need for strict preoperative fasting has also been recently questioned. Most clinical practice guides suggest a period of absolute fasting of between two and six hours, but recent studies indicate that taking a carbohydrate-rich drink before surgery may reduce the endocrine catabolic response and improve insulin resistance [3,16], improving surgical results and hastening recovery. The present ERAS included the administration of carbohydrate-rich drinks (4 × 200 ml) one day prior to surgery plus two further such drinks (2 × 200 ml) on the morning of surgery. The protocol also included the administration of goal directed fluids made possible by the standard use of oesophageal Doppler monitoring [3,17-21], temperature control to avoid hypothermia [3], and the non-routine use of a nasogastric tube; meta-analyses of several trials suggest the latter may reduce pulmonary complications [3,22]. A further measure was the avoidance of routinely using drainage; several randomised trials have suggested that drainage is of no benefit [3,23,24]. Drainage can be avoided in most patients or limited to a short period, facilitating early mobilisation [3], a measure also called for by the ERAS followed. Finally, although taking food orally is commonly limited in the postoperative period, a number of studies have shown that it is safe even after colon surgery involving anastomosis [3,25,26]; it was therefore included in the present ERAS.

Additional variables not explicitly included in the ERAS were also measured: use of prophylactic medication for postoperative vomiting and nausea, use of epidural anaesthesia [3], and opioid-free pain control, it has been reported that opioid-free or opioid-reduced analgesia may hasten recovery [3].

When protocols such as the present are implanted, the goal is that there should be full compliance with all measures outlined. However, full compliance is commonly very difficult to achieve [12]. In the present work the overall compliance rate was 65%, but varied widely in its different components. Patients received information in nearly all cases, while compliance with the provision of early postoperative fluids seemed particularly difficult.

The items of the protocol with less compliance were early oral fluid administration, goal-directed fluid therapy and early mobilization. The reason why these items obtained different compliance with the protocol could be the taste of oral fluid, rejection by patients, unavailability of devices and temporary employment of some healthcare providers involved in the ERAS. Probably the implementation could improve involving and training all the professionals who assist the patients included in the protocol and identifying these patients with signboards on bedside.

Missing values were most important for the variable surgical approach with 6.2% of them, being lower in the other variables.

The rates of complications and mortality recorded were similar to those reported by other authors in randomized controlled trials [5,8-11]. In the present work the most common complication was wound infection. These programmes do not, therefore, appear to place the patient at any extra risk. Our results are similar to previous multicenter studies [27,28] in terms of surgical complications, mortality and readmission rate. The surgical complication rate was 24% compared to the 14.1% reported by Schwenk et al [27] and 20% reported by Braumann et al [28]. The mortality rate was 1% compared to the 0.8% reported by Schwenk et al [27] and 0,4% reported by Braumann et al [28]. The readmission rate was 2.7% compared to the 3.9% reported by Schwenk et al [27] and 4% reported by Braumann et al [28].

Finally, the use of the present ERAS was associated with a preoperative hospital stay of fewer than 24 h and an overall mean stay of 6 days. In other recently published Spanish multicenter study, including data from 50 hospitals, the mean postoperative stay after colorectal resection was 12.36 days [29].

## Conclusion

The present ERAS posed no risk to patients in terms of morbidity, mortality and shortened their hospital stay. The present results show that these programmes can be of benefit to patients and, by reducing hospital costs, may benefit society as a whole.

## Competing interests

The authors declare that they have no competing interests.

## Authors' contributions

Design: JMR, JVR, JEC, FE, JAB, DC and SM.

Data collection: JMR, JVR, JEC and FE.

Data analysing: SM, DC, JAB and JMR.

Writing of manuscript: SM, JAB, JMR and DC.

All authors have read and approve the final manuscript.

## Pre-publication history

The pre-publication history for this paper can be accessed here:

http://www.biomedcentral.com/1471-2482/11/9/prepub
